# Brain Natriuretic Peptide and Troponin T in Patients With Acute Pulmonary Embolism and Grade 3 Obesity: A Retrospective Analysis

**DOI:** 10.7759/cureus.9265

**Published:** 2020-07-19

**Authors:** Carolina Borz-Baba, Mian Munir, Dorothy Wakefield, Richard Feinn

**Affiliations:** 1 Internal Medicine, Saint Mary's Hospital, Waterbury, USA; 2 Internal Medicine, Saint Mary’s Hospital, Waterbury, USA; 3 Statistics, Saint Francis Hospital & Medical Center, Hartford, USA; 4 Statistics, Frank H. Netter M.D. School of Medicine, North Haven, USA

**Keywords:** biomarker, prognosis, pulmonary embolism, grade 3 overweight

## Abstract

Introduction

The interpretation of brain natriuretic peptide (BNP) and Troponin T (TnT) in patients with obesity is very challenging. The applicability of these biomarkers as prognostic indicators of increased mortality in pulmonary embolism (PE) in patients with Grade 3 obesity has yet to be determined.

Methods

To investigate whether the combination of BNP and TnT may help to identify patients at low risk for short-term mortality, we assessed 92 patients admitted with the diagnosis of PE and Grade 3 obesity. The study endpoint was all-cause mortality at 30 days.

Results

The negative predictive value (NPV) of these tests combined is 98.8%; however, we were not able to detect a statistically significant difference between the patients who had a BNP < 100 pg/mL and TnT < 0.03 ng/mL and the other individuals who had either BNP ≥ 100 pg/mL or TnT ≥ 0.03 or both. The mortality rate was 5.43% within 30 days of the diagnosis. The logistic regression analysis using BNP and troponin as continuous variables identified BNP (p < 0.005) as an independent predictor for 30 days mortality. Receiver operating characteristic (ROC) analysis determined that a BNP level of 684 pg/mL was the cutoff level to predict mortality in the population studied.

Conclusions

Our results support that BNP and TnT levels retain an excellent NPV among patients with PE and Grade 3 obesity. BNP testing could be an independent predictor of high-risk patients in this population. The low incidence of all-cause mortality in this study (5.43%) is primarily explained by the more frequent use (9.75%) of systemic or catheter-based thrombolysis associated with a lower rate of major bleeding compared to the general population.

## Introduction

Pulmonary embolism (PE) is currently the third most common acute cardiovascular disease worldwide with a 10-30% estimated mortality within 30 days of diagnosis [1,2]. With an age-adjusted prevalence of 42.45%, obesity has emerged amongst the common modifiable provoking factors for PE [3]. When employed in the appropriate clinical circumstances and in conjunction with imaging findings, the markers of myocardial injury (cardiac troponins, I or T) and right ventricular dysfunction (BNP) are considered essential in the risk stratification of patients with pulmonary embolism. The presence of lower BNP values in obese patients and of a linear increase in high sensitivity troponin T level in patients with a BMI > 35 kg/m^2^ poses a challenge in the accurate interpretation of these biomarkers in patients with acute pulmonary embolism and Grade 3 obesity (BMI ≥ 40 kg/m^2^) [4,5]. The role of concomitant use of both cardiac markers in the prognosis of mortality among patients with obesity Grade 3 has not been studied yet.

## Materials and methods

Study objectives

Clinical and biochemical data were selected via electronic medical record (EMR) chart review from all consecutive admissions with the diagnosis of acute pulmonary embolism and Grade 3 obesity between 2016 and 2019 (July 2016-December 2019) from three hospitals in the region. We included patients with a concomitant diagnosis of congestive heart failure, chronic obstructive pulmonary disease (COPD), renal failure, malignancy, hemodynamic instability and patients who received thrombolysis. We retrospectively searched for all consecutive assays for cardiac-T and BNP of these subjects.

Biochemical assay: Samples were analyzed using different types of quantitative methods based on the institution’s medical equipment. BNP was assessed by immunoenzymatic method, Beckman, or immunofluorescence method by TRIAGE. The cut-off value for normal BNP was <100 pg/mL. Troponin T (TnT) was analyzed by immunoenzymatic, Becker, or immunofluorescence method by TRIAGE. The cut-off for abnormal TnT was ≥0.03 ng/mL.

To determine the predictive value of both BNP and troponin, the participants in the study were divided into four groups. Group 1 of subjects had a normal BNP (defined as <100 pg/mL) and normal TnT (<0.03 ng/mL), group 2 of subjects had high BNP (≥100 pg/mL) and normal TnT (<0.03 ng/mL), group 3 of patients had at least one abnormal TnT (≥0.03 ng/mL) and normal BNP (<100 pg/mL) and group 4 of subjects with high BNP (≥100 pg/mL) and at least one abnormal TnT (≥0.03 ng/mL). Radiological evidence of right ventricular dysfunction, defined by computed tomography pulmonary angiography (CTPA) (right ventricular diameter/left ventricular diameter ratio of ≥1 in conjunction with septal bowing) was examined for the patients in group 1. The imaging was reviewed independently by two radiologists. Hemodynamic instability was defined as systolic blood pressure (SBP) < 90 mmHg for at least 15 minutes.

Study endpoint

The endpoint of the study was all-cause mortality within 30 days of diagnosis. The protocol of the study was approved by the IRB committee.

Statistical analysis

Data categorized by a normal distribution are represented as mean values followed by the standard deviation. For the categorical variables, we used Wald Chi-Square. Logistic regression was performed for the set of univariable predictors. The tests used included a two-sided analysis. Results were considered statistically relevant at P < 0.05. We used Statistical Analysis System software for the statistical calculations.

## Results

Demographics

Acute pulmonary embolism was diagnosed in 130 patients with Grade 3 obesity. The diagnosis was based on clinical evidence and imaging data obtained by CTPA or ventilation-perfusion (V/Q) scan. Echocardiography was employed to determine if right ventricular dysfunction (RVD) was present in 90.7% of cases. Only 92 patients (70.7%) had both BNP and at least one TnT assessed during hospitalization and were finally enrolled in the study.

The study included 68 (74%) women and 24 (26%) men aged: 55 +/- 14 SD with BMI 47 +/- 7 SD. At least one of the following coexisting conditions was present in 44 patients (47.8%): congestive heart failure (CHF), renal failure (RF), coronary artery disease (CAD), chronic obstructive pulmonary disease (COPD) and malignancy. Hemodynamic instability, defined as a systolic blood pressure of <90 for at least 15 minutes, was documented in 14/92 (15.2%). Either systemic or catheter-based thrombolysis was administered in nine cases (9.75%), and no patients underwent thrombectomy. None of the patients who received thrombolysis expired in the first 30 days. Death was recorded in five patients (5.43%) included in the study; however, six patients (4.61%), of the total number of 130 patients initially screened, died at 30 days. Three patients presented more than once with the diagnosis of acute pulmonary embolism, but none expired in the first 30 days.

The group characteristics based on the BNP and troponin levels are detailed in Table 1.

**Table 1:** Characteristics of the patients by group BNP: Brain natriuretic peptide; TnT: Troponin T; BMI: Body mass index; SBP: Systolic blood pressure; CHF: Congestive heart failure; CAD: Coronary artery disease; COPD: Chronic obstructive pulmonary disease.

	Group 1 BNP < 100 pg/mL, TnT < 0.03 ng/mL	Group 2 BNP ≥ 100 pg/mL, TnT < 0.03 ng/mL	Group 3 BNP < 100 pg/mL, TnT ≥ 0.03 ng/mL	Group 4 BNP ≥ 100 pg/mL, TnT ≥ 0.03 +ng/mL
Number of patients	25	6	24	37
Age	52 +/- 13	58 +/- 15.5	52.8 +/- 14	57.7 +/- 13.3
Female gender n (%)	19 (76)	4 (66)	15 (62.5)	23 (62)
BMI	45.7 +/- 5	52.3 +/- 13	47 +/- 5.8	47.1 +/- 7.6
SBP < 90 mmHg n (%)	0 (0)	2 (33)	1 (4)	12 (32)
CHF n (%)	5 (20)	5 (83)	5 (21)	12 (25.5)
CAD n (%)	0 (0)	2 (33)	1 (4)	6 (16)
COPD n (%)	3 (12)	0 (0)	3 (12.5)	6 (16)
Renal failure n (%)	3 (12)	2 (33)	8 (33)	14 (38)
Malignancy n (%)	5 (20)	1 (17)	3 (12.5)	2 (5)
All cause mortality n (%)	0	0	1 (4)	4 (11)

The negative predictive value of combined BNP <100 and TnT <0.03 with a 95% CI is 98.8%. PPV of BNP ≥ 100 and TnT ≥ 0.03 is 10.8%.

**Figure 1:**
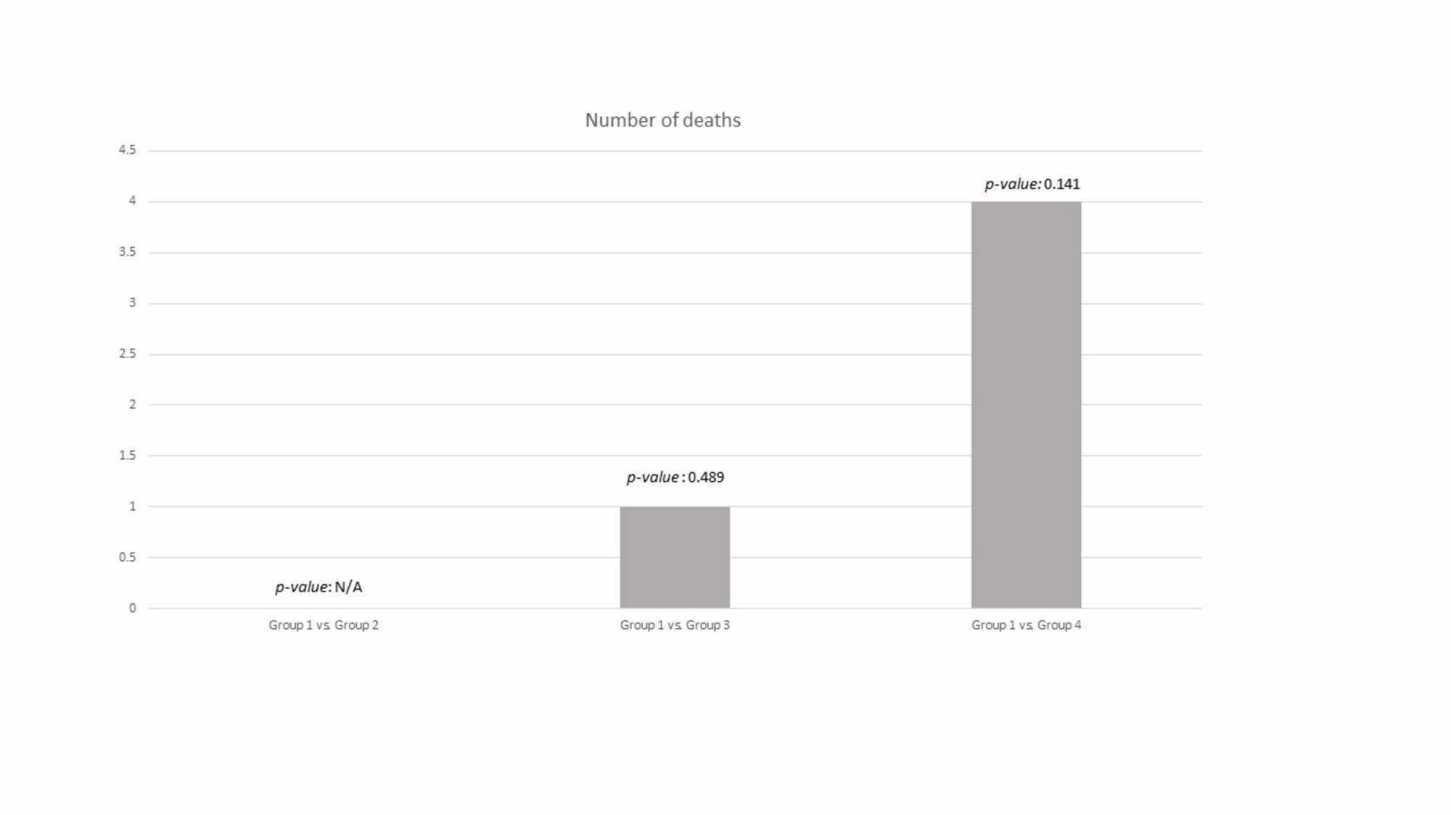
Primary endpoint between groups using a brain natriuretic peptide (BNP) cutoff of 100 pg/mL

**Figure 2:**
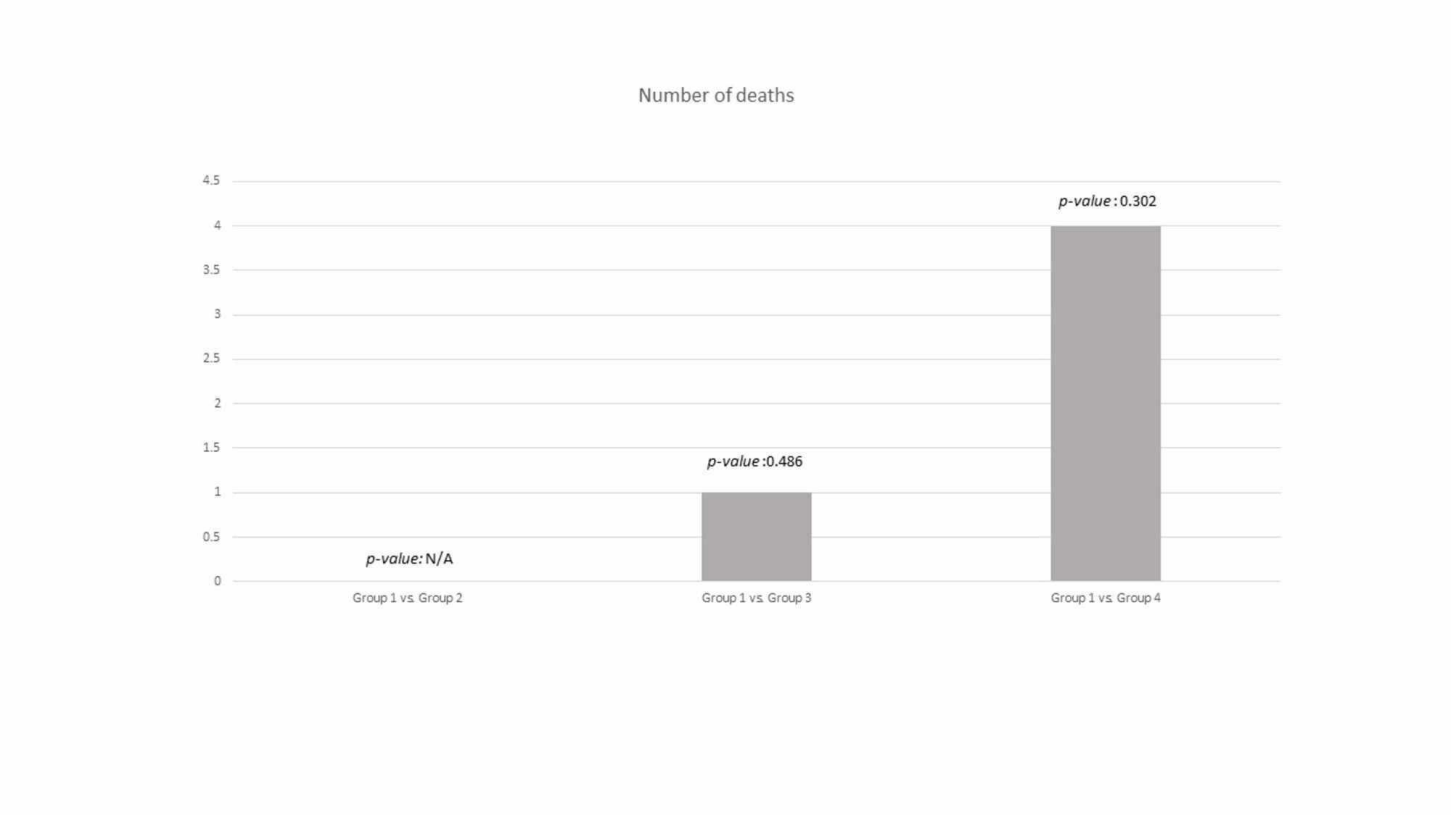
Primary endpoint using a brain natriuretic peptide (BNP) cutoff of 54 pg/mL

When comparing group 1 with the other groups, using either a BNP cutoff of 100 pg/mL (Figure 1) or 54 pg/mL (Figure 2), no statistical difference was noted.

The Fisher’s exact test using BNP and TnT as continuous variables determined that only BNP is the statistically significant predictor for mortality among patients with obesity Grade 3 admitted for acute PE (Table 2).

**Table 2:** Logistic regression of variables BNP: Brain natriuretic peptide; TnT: Troponin T; CHF: Congestive heart failure; COPD: Chronic obstructive pulmonary disease; CAD: Coronary artery disease.

Variables	OR (95% CI)	p-value
Age ≥ 55	3.57 (0.38, 33.20)	0.3709
Gender, F vs M	2 (0.21, 18.7)	1.000
BNP	1.003 (1.001, 1.005)	0.0022
Maximum TnT	0.92 (0.57, 1.68)	0.6848
CHF	3.93 (0.61, 25.04)	0.148
COPD	1.06 (1.00, 1.12)	1.00
Renal failure	3.93 (0.619, 25.04)	0.148
CAD	2.46 (0.24, 24.84)	0.4096
Malignancy	1.92 (0.195, 18.97)	0.479

**Figure 3:**
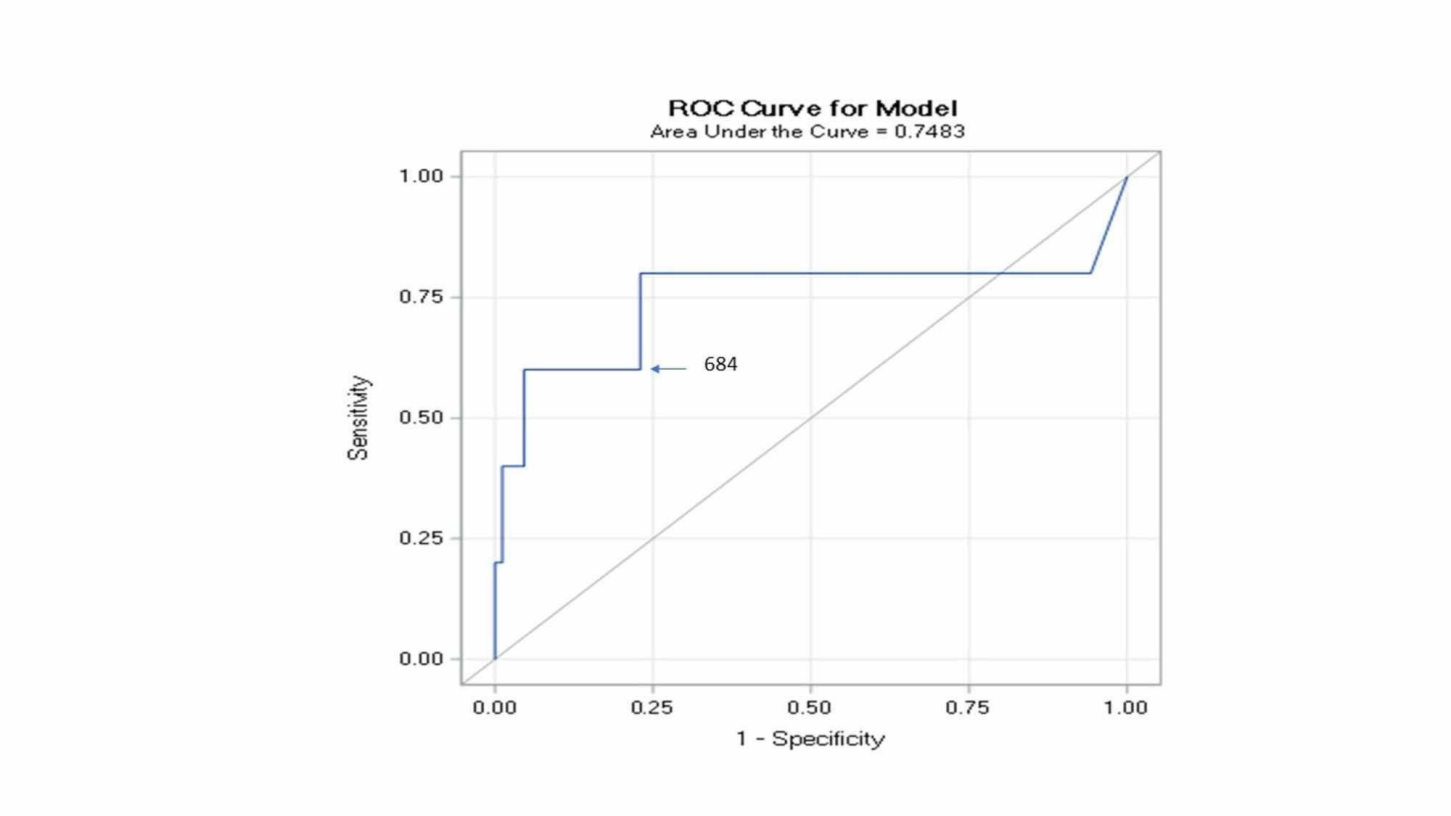
ROC analysis

Receiver operating characteristic (ROC) analysis (Figure 3) identified that a level of 684 (sensitivity of 60%, specificity of 93.1%) is the cutoff level to predict mortality in the population studied.

## Discussion

Obesity is increasing considerably worldwide. Based on an average prevalence of obesity of 35.7% [6], the incidence of venous thromboembolism of 1.92/1000 cases [2] and a likelihood of venous thromboembolism (VTE) among obese population almost twice that of persons without obesity (OR of 1.84 (95% CI, 1.55-2.18)) [7], we estimated that between 2009-2010, in a population with ages between 45 and 79, like ours, the incidence of VTE was 2.7/1000 cases. In the general population, 60% of all VTE occurs in people aged 70 or older, but patients with obesity Grade 3 have a tendency to experience first pulmonary embolism at a much younger age [8]. The mean age in our study was 55 SD +/- 14 years, similar to the mean age reported in other studies 57 +/- 17 years [9]. According to our demographics, there is a higher risk of PE in Grade 3 obesity women, as determined by others, who recognized that the relative risk of idiopathic PE (defined as subjects without a history of surgery or major trauma in the last month) was as high as 5.79 in individuals with a BMI ≥ 35 kg/m^2^ (p < 0.001) [10].

Hemodynamic instability, defined as SBP < 90 mmHg for at least 15 minutes, is a critical determinant of prognosis and the main clinical indicator to administer the definitive treatment represented by systemic or catheter-based thrombolysis or thrombectomy. Hemodynamic instability was documented in 15.2% of cases, with the majority recorded among the patients in group 4. All patients in group 1 (100%) and 96% of patients in group 3 had a sustained SBP ≥ 90 mmHg.

In acute PE, BNP is released due to right ventricular wall distension as a consequence of pressure overload and right ventricular dysfunction (RVD). A recent systematic review and meta-analysis examined the prognostic value of BNP in the general population with PE and concluded that while a high BNP has a positive predictive value of around 14% (95 CI 11% to 18%), a normal BNP has a negative predictive value (NPV) of 99% (CI 97% to 100%) [11]. Troponin I and T are markers of myocardial injury. According to a recent metanalysis, an increased troponin is correlated with higher mortality, but a negative high sensitivity troponin level provides an NPV of 98% of prediction a 30-day outcome in the patients with PE [12,13].

The interpretation of both BNP and TnT values in patients with obesity grade 3 is particularly challenging. The majority of our patients (53.2%) had a BNP <100 pg/mL. From the data available in patients with HF, one has to consider the potential falsely low results of BNP in at least 20% of obese subjects [14]. Our assessment confirms that in extremely obese patients, a BNP <100 pg/mL is recorded in 20% of patients with PE, and a coexisting diagnosis of CHF. Up to 66% of individuals had abnormal TnT (≥0.03), but only 8.6% of patients had known CAD. The potential major confounding factor in the troponin interpretation is renal failure, which was present in 36% of patients with abnormal TnT.

Our study’s primary focus was the association between combined normal BNP and normal troponin (Group 1) and mortality at 30 days. All patients in group 1 remained hemodynamically stable throughout the hospitalization. None of them had RVD as defined by CTA (RV diameter/left ventricular diameter ratio of ≥1 in conjunction with septal bowing). The imaging was reviewed independently by two radiologists. Although the NPV of these tests is 98.8%, we were not able to detect a statistically significant difference between group 1 and the other groups in predicting mortality at 30 days. Using a BMI-related BNP cutoff level of 54 pg/mL, recommended by the Breathing Not Properly trial, no additional benefit was noted [15]. The most likely reason for these results was the reduced incidence of all-cause mortality within 30 days (5.43%), which is much lower than previously reported, likely owing to the immediate reperfusion strategies employed in these cases and much younger age than the general population with PE [2]. Either systemic or catheter-based thrombolysis in our study was administered in 9.75% of cases, which is much higher than the number of patients in the general population that benefit from thrombolysis (1.9%-4.2%) [16,17].

The logistic regression analysis, using BNP and troponin as continuous variables, isolated BNP as an independent predictor of mortality at 30 days. ROC analysis determined that a level of 684 is the cutoff level to predict mortality in the population studied. We consider different hypotheses to explain this result. One of the reasons could be that BNP is higher in women, and our population included women predominantly; however, the logistic regression indicated that there was no significant consequence of gender on the risk of mortality. The other potential explanation would be that BNP is a nonspecific biochemical marker that indicates multiple risk factors that reflect ventricular dysfunction [18]. Few reports that compared BNP and troponin in the risk stratification of PE in the general population reached a similar conclusion [19-20]. The absence of association between elevated troponins and outcome is partly due to the low 30-day mortality and the possibility that troponins are frequently abnormal among patients with Grade 3 obesity [5].

Our report included a larger number of patients, but the low rate of all-cause mortality events at 30 days and the retrospective nature of our study make it very difficult to draw further conclusions as to the other associated risk factors.

There are a few more observations that we learned from reviewing the data. In our study, a definitive treatment, such as either systemic or catheter-based thrombolysis, was administered in 9.75% of cases, a much higher figure than the number of patients in the general population that benefit from thrombolysis (1.9% - 4.2%) [16,17]. The main concern following the administration of thrombolytics is significant bleeding, defined by prior studies as intracranial bleed or major bleed requiring transfusions within one week of treatment [21]. In our study, the survival rate was 100% within 30 days with no significant bleeding recorded between treatment and day 14. The presence of comorbidities were higher (47.8%) than in similar studies of non-severely obese subjects, which agrees with the prior observation that advanced age and not the comorbidities is the most important determinant of the bleeding complication related to thrombolysis [22].

## Conclusions

The study provided the opportunity to appreciate a more frequent use of thrombolysis and a low rate of major complications in patients with PE who have Grade 3 obesity. To our knowledge, this is the first report that examined the biomarkers’ behavior in this selected population and estimated the independent predictive value of BNP and troponin but did not include parallel imaging, which is the common practice. The hypothesis that BNP has a higher predictive value than troponin emphasizes the need for prospective validation of the role of the biomarker in the risk stratification in patients with PE and Grade 3 obesity.
